# The Imbalance of MMP-2/TIMP-2 and MMP-9/TIMP-1 Contributes to Collagen Deposition Disorder in Diabetic Non-Injured Skin

**DOI:** 10.3389/fendo.2021.734485

**Published:** 2021-10-27

**Authors:** Peng Zhou, Chao Yang, Shan Zhang, Zun-Xiang Ke, Dian-Xi Chen, Yi-Qing Li, Qin Li

**Affiliations:** Department of Vascular Surgery, Union Hospital, Tongji Medical College, Huazhong University of Science and Technology, Wuhan, China

**Keywords:** MMPs (metalloproteinases), collagen, skin, diabetes, TIMPs

## Abstract

The importance of the early diagnosis and treatment of diabetes and its cutaneous complications has become increasingly recognized. When diabetic non-injured skin was stained with Masson’s trichrome, its dermal collagen was found to be disordered, its density was variable, and it was dispersed or arranged in vague fascicles. The collagen type I sequencing results of RNA sequencing-based transcriptome analysis of three primary human skin cell types—dermal fibroblasts, dermal microvascular endothelial cells, and epidermal keratinocytes—under high glucose were analyzed. The results showed that both COL1A1 and COL1A2 mRNA expressions were reduced in human dermal fibroblasts (HDFs). The ratio of matrix metalloproteinase (MMP)-2/tissue inhibitors of metalloproteinase (TIMP)-2 and MMP-9/TIMP-1 in HDFs increased when treated with high glucose. By inhibiting MMP-2 and MMP-9 with SB-3CT, collagen deposition disorder of the skin in streptozotocin-induced diabetes mice was alleviated. The imbalance of MMP2/TIMP2 and MMP9/TIMP1 contributes to the non-injured skin disorder of collagen deposition in diabetes, suggesting a possibility for early treatment of diabetes skin complications.

## Introduction

Diabetes mellitus (DM) is a metabolic disease caused by multiple etiologies and characterized by chronic hyperglycemia. Further increases in morbidity and mortality from complications of DM are predicted (1). Diabetic skin lesions (2), especially diabetic foot, are of particular concern as they not only reduce the quality of life of patients but also put enormous economic pressure on national healthcare systems. In recent years, the importance of early diagnosis and treatment of diabetes and its complications has become increasingly recognized (3). A series of changes in the skin of diabetes patients before skin injury occurs have aroused great interest in researchers (4). Non-injured skin in diabetes patients may be the basis of skin lesions, and early detection and rapid intervention may reduce or delay the occurrence of skin lesions to a certain extent.

The skin has three layers: epidermis, dermis, and subcutaneous tissue. The integrity of the epidermis and stability of the dermis are the basis for preventing the invasion of external harmful substances. Diabetes damages the structure of dermal collagen, and studies have shown that a higher average score for skin structural defects (5) and inferior biomechanical properties (6) increase the risk for developing DM skin complications. Collagen is the main component of the dermal extracellular matrix (ECM), primarily secreted by fibroblasts and regulated by the balance of matrix metalloproteinases (MMPs) and tissue inhibitors of metalloproteinases (TIMPs) (7, 8).

MMPs are a family of zinc-dependent endopeptidases that are the most important enzymes for the degradation of the ECM. They play a vital role in both physiological and pathological tissue remodeling. TIMPs are endogenous specific inhibitors of MMPs; for instance, TIMP-2 can inhibit the activity of MMP-2 and TIMP-1 can inhibit that of MMP-9 (9). A puncture biopsy of the wound tissue of a chronic ulcer of diabetic skin showed that the expression of MMP-1, -2, -8, and -9 was increased and that of TIMP-2 was decreased (10). The degradation of ECM regulated by MMPs/TIMPs is a crucial cause of poor wound healing in diabetes patients (11). Although it takes a long time for diabetic skin to be injured, the skin tissue will have been impacted by high glucose levels for a long time, and its structure will have largely changed at an early stage (12). The function of the MMP/TIMP balance has been studied in diabetic poor wound healing, but whether this balance contributes to early changes in diabetic skin is unknown. Therefore, it is of great significance to improve the understanding of early non-injured skin changes in diabetes patients, and early intervention is important for the clinical prevention and treatment of skin lesions.

In the current study, we found that dermal collagen deposition disorder occurred in the skin of some diabetes patients before evident skin injury. Moreover, the cause of the changes in the non-injured skin collagen targeting the balance of MMP2/TIMP2 and MMP9/TIMP1 was investigated.

## Materials and Methods

### Acquisition and Preparation of Human Skin Tissues

A total of 10 participants comprising five concomitant DM subjects and five control subjects without diabetes participated in this study. Skin tissues were collected from the patients during surgery. The skin samples were fixed in 4% paraformaldehyde after being harvested. Dehydration in acetone was followed by embedding in paraffin. The project was approved by the Wuhan Union Hospital Ethics Committee (REC 08/H1202/137). All participants provided written informed consent.

### Cell Culture and Sample Preparation

All steps for cell culture were consistent with those of our previous study (10). Briefly, human dermal fibroblasts (HDFs; Cat # 2320, ScienCell) were cultured in normal glucose (8 mM) and high glucose (30 mM) media for 24 h, respectively, which is wildly employed to simulate diabetic conditions in numerous published studies (13, 14). Total cellular RNA was extracted according to a standard TRIzol RNA extraction protocol using RNAiso Plus (Cat # 9108, Takara). Moreover, total proteins from the cells were extracted by RIPA lysis buffer (Cat # P0013K, Beyotime). For cell immunohistochemistry analysis, cells were cultured on 24-mm cover slips (Cat # YA0352, Solarbio).

### Animals

Specific pathogen-free C57BL/6 mice (male, 6–8 weeks old) weighing 23 ± 2 g were purchased from SHULAIBAO Biotech. (Hubei, China). The animals were housed in conventional animal facilities in a temperature- and humidity-controlled environment with a 12-h light/dark cycle. A standard or 60 kcal% fat (Cat # D12492, HFK Bioscience) diet was provided. All animals received care in compliance with the Principles of Animals Use Committee (NIH Publications No. 8023, revised 1978).

### Study Design, Diets, Treatments, and Type 2 Diabetes Mouse Model

A high-fat diet combined with one-time pharmacological intervention of streptozotocin (STZ) was used to create a type 2 diabetes (T2D) model, which is considered suitable for inducing hallmark features of human T2D (15). The mice were divided into four groups (*n* = 7 each): high-fat diet combined with STZ followed by SB-3CT (HFD/STZ-SB, STZ dissolved in Na-citrate solution, SB-3CT dissolved in DMSO), HFD/STZ combined with DMSO (HFD/STZ-DMSO), normal diet with solvents (Normal), and normal diet with SB-3CT (Normal-SB). After 12 h of fasting, STZ (120 mg/kg, Cat # S1312, Selleck) was administered *via* an intraperitoneal injection after a high-fat diet for 4 weeks. SB-3CT (10 mg/kg, Cat # S7430, Selleck), which is widely prescribed in previous studies (16–18), was injected every other day for 3 weeks starting the day after STZ administration. During this period, a high-fat diet was continued, and nonfasted glucose measurements were obtained every 5 days from the blood of the tail, after which euthanasia was performed followed by back skin tissue harvesting.

### RNA Sequencing

The RNA-seq data were obtained from our previous study and have been uploaded to a public database (https://db.cngb.org/search/project/CNP0000999/) (19).

### Real-Time Quantitative PCR

The extracted RNA was reverse-transcribed into cDNA using a cDNA synthesis kit (Cat # RR037A, Takara). Real-time quantitative PCR was implemented on an ABI StepOne Plus System (Applied Biosystems, Foster City, CA) using SYBR Premix Ex Taq (Cat # RR420A, Takara). The primers used were as follows: MMP-2, 5’-TACAGGATCATTGGCTACACACC-3’ (forward) and 5’-GGTCACATCGCTCCAGACT-3’ (reverse); TIMP-2, 5’-TCTCGACATCGAGGACCCAT-3’ (forward) and 5’-TGGACCAGTCGAAACCCTTG-3’ (reverse); MMP-9, 5’-TGTACCGCTATGGTTACACTCG-3’ (forward) and 5’-GGCAGGGACAGTTGCTTCT-3’ (reverse); and TIMP-1, 5’-TCCAAGGCTCTGAAAAGGGC-3’ (forward) and 5’-ATTCAGGCTATCTGGGACCG-3’ (reverse). All primers were purchased from Sangon Biotech. The mRNA levels of the target genes were normalized to GAPDH using the 2^−ΔΔCT^ method.

### Western Blot

The extracted proteins were separated using 10% SDS electrophoresis before transfer onto a nitrocellulose membrane. The membrane was separately probed with a respective primary antibody (MMP-2, Cat # A11144; TIMP-2, Cat # A1558; MMP-9, Cat # A0289; TIMP-1, Cat # A1389, ABclonal) for 8 h at 4°C followed by incubation with horseradish peroxidase-labeled secondary antibody for 2 h at 37°C. Enhanced chemiluminescence reagent (Cat # MA0186, Meilunbio) was then added to the blots and the bands were analyzed using ImageJ software (NIH, USA).

### Cell Immunohistochemistry

Coverslips were washed with TBS, fixed with 4% paraformaldehyde for 15 min, and permeabilized with 0.1% Triton X‐100 for 10 min. The coverslips were treated with the primary and secondary antibodies (Cat # ANT058, antGene) and then visualized with DAB (Dako) and counterstained with hematoxylin, before being analyzed by light microscopy.

### Masson’s Trichrome Staining

All staining of both human and mouse samples was conducted by Biossci Company (Hubei, China) with a custom-designed kit (Cat # BP028). The skin samples were fixed in 4% paraformaldehyde. Dehydration in acetone was followed by embedding in paraffin. Ultrathin sections were stained with phosphomolybdic acid and toluidine blue. Thereafter, collagen fiber alignment was determined from images captured using a microscope.

### Statistical Analysis

Data are presented as the mean ± SEM. Normally distributed quantitative variables were compared using Student’s *t*-test. Values of *p* < 0.05 were considered statistically significant. All figures were prepared using GraphPad Prism 7.0 and Adobe Illustrator software.

## Results

### Disordered Collagen Deposition and Decreased Collagen Expression in Human Diabetic Skin

**Figure 1:**
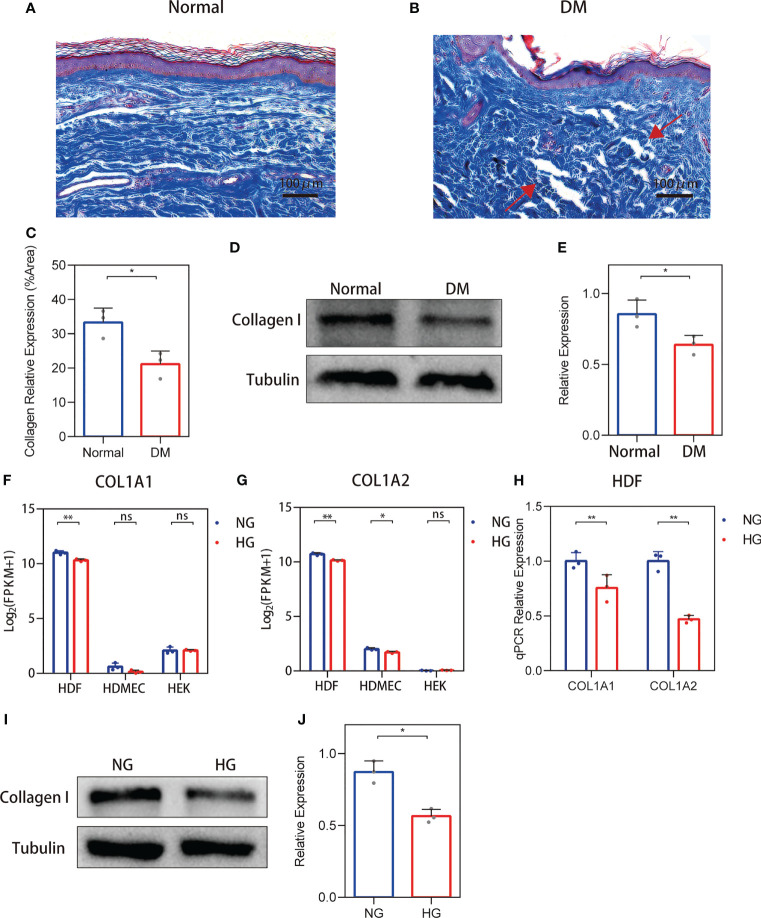
Changes in the collagen deposition and expression of diabetic skin. **(A, B)** Representative Masson’s trichrome stain of non-diabetic (*n* = 6) and diabetic skin (*n* = 7). **(C)** The quantitative analysis of collagen stain. **(D, E)** Western blot and their quantitative analysis of collagen I expression in skin. **(F, G)** COL1A1 and COL1A2 mRNA expressions in three primary human skin cell types—human dermal fibroblasts (HDFs), human dermal microvascular endothelial cells, and human epidermal keratinocytes—cultured with high glucose. **(H)** RT-qPCR verification of the RNA-seq data. **(I, J)** Western blot and their quantitative analysis of collagen I expression in HDFs cultured with high glucose. The results are provided as the means ± SEM, *p < 0.05, **p < 0.01 and ‘ns’ means no significance compared with the control.

For non-diabetic skin tissue, close to the plane of the epidermis, collagens (indicated in blue) show a fine bundle-like morphology, with chiefly thick bundles arranged close to the subcutaneous tissue. These collagens were uniformly colored and tightly arranged (**Figure 1A**). In the diabetic skin, collagen was disordered and density was variable, and it was dispersed or arranged in vague fascicles (**Figure 1B**). Collagen staining quantification (**Figure 1C**) and Western blot (**Figures 1D, E**) results show that the expression of collagen in diabetic skin was decreased.

### Collagen I Expression Was Reduced in HDFs With High Glucose Stimulation

To clarify the source of cells causing changes in skin collagen, the collagen type I sequencing results of RNA-seq-based transcriptome analysis of three primary human skin cell types—HDFs, dermal microvascular endothelial cells, and epidermal keratinocytes—under high glucose were analyzed according to the methods of our previous study (19). RNA-Seq results showed that both COL1A1 and COL1A2 mRNA expressions were reduced in high glucose-treated HDFs (**Figures 1F, G**). RT-PCR verification of the RNA-seq data show that both the expression of COL1A1 and COL1A2 were decreased (**Figure 1H**). In addition, the protein levels of collagen I in high glucose-treated cell cultures showed a decrease (**Figures 1I, J**). RNA-seq and qPCR analysis of the MMP-2/TIMP-2 and MMP-9/TIMP-1 ratios in high glucose-treated HDFs showed an increase.

### RNA-Seq and qPCR Analysis of the MMP-2/TIMP-2 and MMP-9/TIMP-1 Ratios in High Glucose-Treated HDFs Showed an Increase

**Figure 2:**
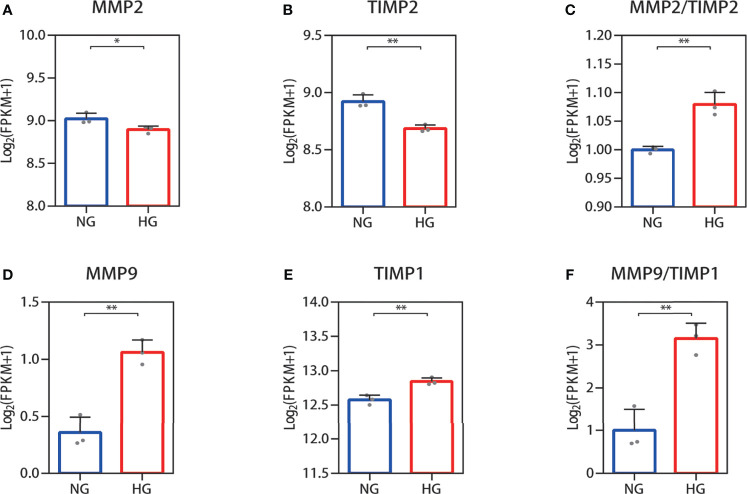
RNA-seq quantification of the MMP-2/TIMP-2 and MMP-9/TIMP-1 ratio in high glucose-treated HDFs. **(A–C)** Quantitative expression profiling of MMP-2 and TIMP-2 mRNA in HDFs and the MMP-2/TIMP-2 ratio. **(D–F)** The expression of MMP-9 and TIMP-1 mRNA in HDFs and the MMP-9/TIMP-1 ratio. The results are provided as the means ± SEM, ^*^ *p* < 0.05, ^**^ *p* < 0.01 compared with the control.

**Figure 3:**
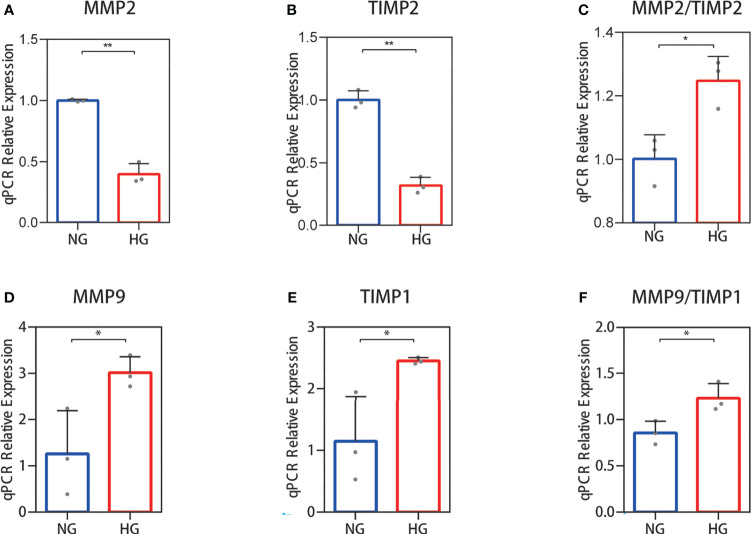
RT‐qPCR validation of the MMP-2/TIMP-2 and MMP-9/TIMP-1 ratio in high glucose-treated HDFs. **(A–C)** The expression of MMP-2 and TIMP-2 mRNA in HDFs and the MMP-2/TIMP-2 ratio. **(D–F)** MMP-9 and TIMP-1 mRNA expression in HDFs and the MMP-9/TIMP-1 ratio. The independent experiment was repeated three times. The results are provided as the means ± SEM, ^*^ *p* < 0.05, ^**^ *p* < 0.01 compared with the control.

Collagen in skin is synthetized primarily by HDFs. The ratio changes of MMP-2/TIMP-2 and MMP-9/TIMP-1, which can regulate synthesis and breakdown of collagen, were detected in HDFs with high glucose *in vitro*. In RNA-seq, the reduced MMP-2 and TIMP-2 mRNA expression levels were observed in HDFs compared with normal glucose (**Figures 2A, B**). The value of MMP-2/TIMP-2 increased (**Figure 2C**). MMP-9 and TIMP-1 expression levels showed an increase, and their ratio (MMP-9/TIMP-1) increased similarly in the high glucose group compared with the normal group (**Figures 2D–F**). For the RNA-seq results, log2 (FPKM + 1) values are shown. Consistent with the RNA-seq results, the qPCR results showed that the MMP-2 and TIMP-2 expression levels were reduced (**Figures 3A, B**), the value of MMP-2/TIMP-2 was increased (**Figure 3C**), and the MMP-9 and TIMP-1 expression levels and MMP-9/TIMP-1 ratio increased (**Figures 3D–F**) in the high glucose group compared with the normal group.

### Western Blot Analysis of the MMP-2/TIMP-2 and MMP-9/TIMP-1 Ratio in High Glucose-Treated HDFs Showed an Increase

**Figure 4:**
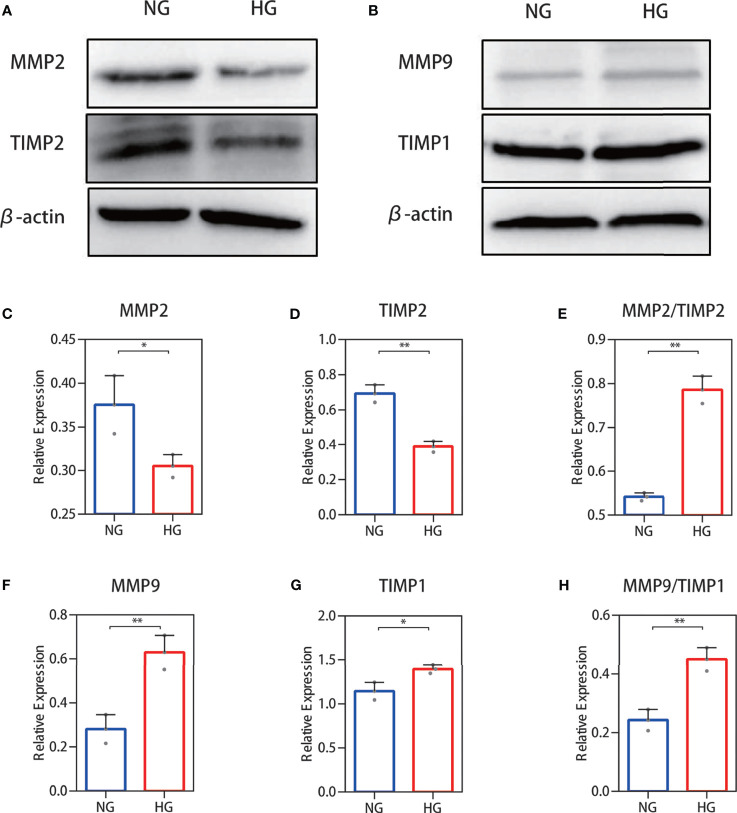
Western blot analysis of the MMP-2/TIMP-2 and MMP-9/TIMP-1 ratio in high glucose-treated HDFs. **(A, B)** Representative Western blots of MMP-2 and TIMP-2 as well as MMP-9 and TIMP-1. **(C, E)** Semiquantitative analysis of MMP-2, TIMP-2, and the MMP-2/TIMP-2 ratio. **(F–H)** Semiquantitative analysis of MMP-9, TIMP-1, and the MMP-9/TIMP-1 ratio. The independent experiment was repeated three times. The results are provided as the means ± SEM, ^*^ *p* < 0.05, ^**^ *p* < 0.01 compared with the control.

To further validate the above results, Western blotting was performed. Protein expression analysis results were in accordance with our other results. Decreased MMP-2 and TIMP-2 protein levels (**Figure 4A**) and increased MMP-9 and TIMP-1 protein levels (**Figure 4B**) were observed in HDFs with high glucose stimulation. Gray value analysis of the WB bands was undertaken and the ratio of MMP-2/TIMP-2 and MMP-9/TIMP-1 was determined, and both showed an increase (**Figures 4C–H**).

### Cell Immunohistochemistry Analysis of the MMP-2/TIMP-2 and MMP-9/TIMP-1 Ratio in High Glucose-Treated HDFs Showed an Increase

**Figure 5:**
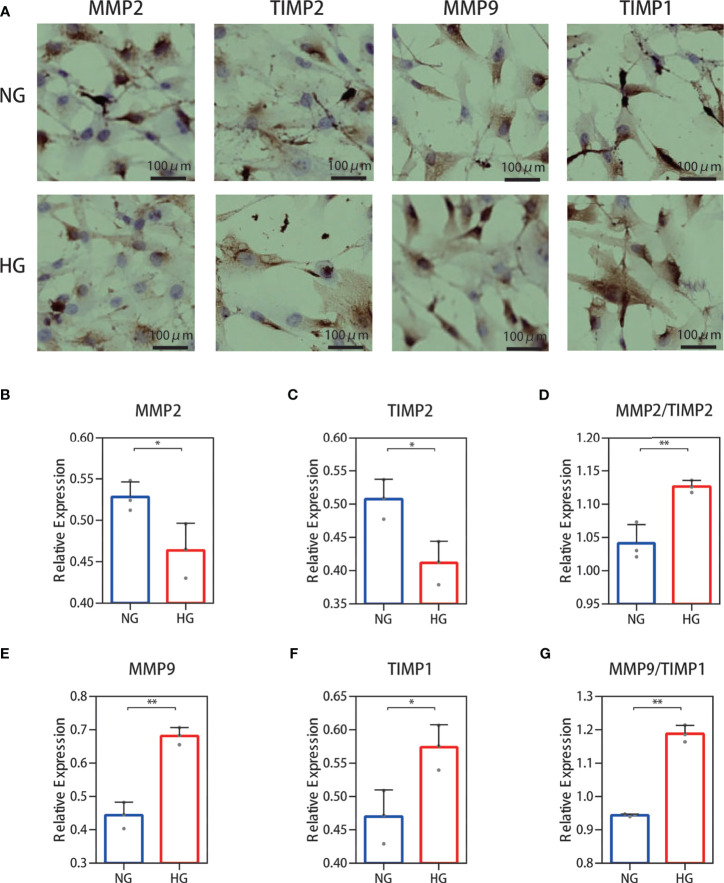
Cell immunohistochemistry stain of the MMP-2/TIMP-2 and MMP-9/TIMP-1 ratio in high glucose-treated HDFs. **(A)** Representative cell staining of MMP-2, TIMP-2, MMP-9, and TIMP-1 in HDFs. **(B–D)** Semiquantitative analysis of MMP-2, TIMP-2, and the ratio of MMP-2/TIMP-2. **(E–G)** Semiquantitative analysis of MMP-9, TIMP-1, and the ratio of MMP-9/TIMP-1. The independent experiment was repeated three times. The results are provided as the means ± SEM, ^*^ *p* < 0.05, ^**^ *p* < 0.01 compared with the control.

The results for protein levels were further validated through cell experiments. We examined the secretions of MMP‐2, TIMP-2, MMP‐9, and TIMP-1 immunohistochemically. The results showed that MMP-2 and TIMP-2 secretion levels were decreased and those of MMP-9 and TIMP-1 were increased (**Figure 5A**) in the high glucose group compared with the normal group. Immunohistochemical staining was quantified using ImageJ software (**Figures 5B–G**) and showed an increase in the ratio of MMP-2/TIMP-2 and MMP-9/TIMP-1.

### Assessment of Animal Models

Overall, 11 of the 14 mice were successfully established as a diabetes model (six from the HFD/STZ-SB group and five from the HFD/STZ-DMSO group), defined as having glucose levels above 14 mmol/L (20, 21). The last measurement of plasma glucose concentration was used to calculate the average value [20.5 (19.3–21.6) mmol/L] of these diabetic mice. During the study, two mice died (one from the HFD/STZ-SB group and one from the Normal-SB group).

### MMP-2 and MMP-9 Inhibitor SB-3CT Decreased the Disordered Collagen Deposition of Skin in STZ-Induced Diabetes Mice

**Figure 6:**
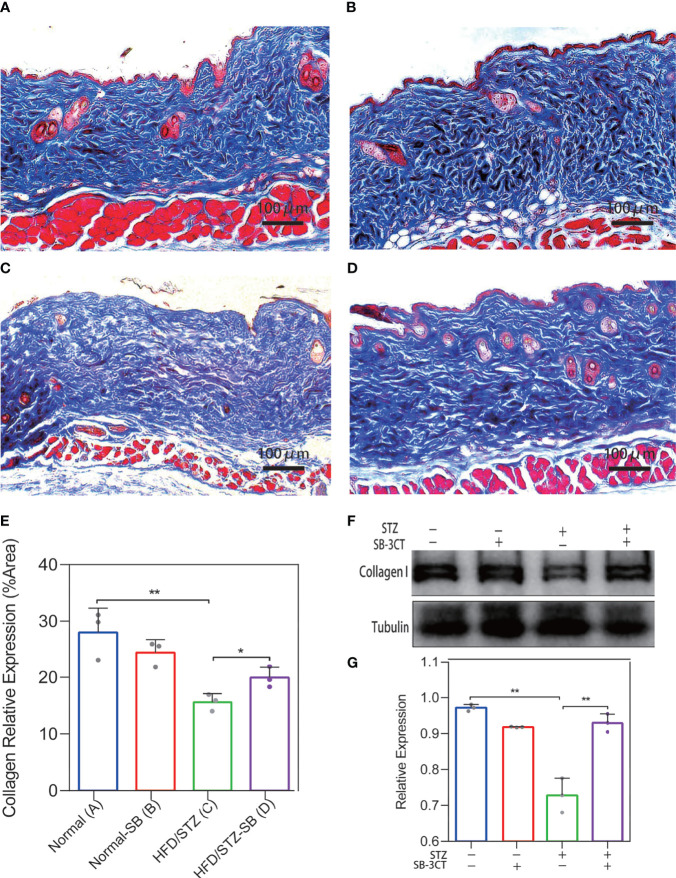
Changes in collagen deposition of STZ-induced diabetic mouse skin with the inhibitor (SB-3CT) of MMP-2 and MMP-9. **(A–D)** Representative Masson’s trichrome stain of skin in the Normal (*n* = 7), Normal-SB (*n* = 6), HDF/STZ-DMSO (*n* = 5), and HDF/STZ-SB groups (*n* = 5). **(E)** The quantitative analysis of collagen stain. **(F, G)** Western blot and their quantitative analysis of collagen I expression in skin of different mice. (Normal group: normal diet with solvent DMSO; Normal-SB group: normal diet with SB-3CT; HDF/STZ-DMSO group: high-fat diet combined with STZ followed by DMSO solvent; HDF/STZ-SB group: high-fat diet combined with STZ followed by SB-3CT.). The results are provided as the means ± SEM, *p < 0.05 and **p < 0.01 compared with the control.

We considered the possibility that the collagen deposition disorder was caused by the imbalance of the MMP-2/TIMP-2 and MMP-9/TIMP-1 ratio. *In vivo*, the ratio of MMP2/TIMP2 and MMP9/TIMP1 was also elevated in the skin of diabetes mice by qPCR analysis (**Supplementary Figure S1**) Therefore, MMP-2 and MMP-9 inhibitors were administered to the STZ-induced diabetic mice to evaluate the changes in skin collagen *in vivo*. In the Masson’s trichrome staining of skin in the Normal (**Figure 6A**) and Normal-SB groups (**Figure 6B**), collagen was uniformly colored and tightly arranged. The skin of the HFD/STZ-DMSO group (**Figure 6C**) was filled with loosely packed and fragmented collagen that was disordered with variable density. Inhibiting MMP-2 and MMP-9 with SB-3CT (HFD/STZ-SB) alleviated skin disordered collagen deposition (**Figure 6D**) compared to HFD/STZ. Similarly, the quantitative results of collagen staining demonstrate that the expression of collagen I was rescued partly after the function of gelatinases MMP-2 and MMP-9 were inhibited in the mouse models of DM (**Figure 6E**). Western blot analyses (**Figures 6F, G**) were conducted, confirming the results of collagen staining.

## Discussion

Dermal collagen deposition disorders in diabetic skin prior to skin injury have increased clinical awareness and caution toward complications of diabetic skin. The collagen deposition disorder can be caused by either decreased synthesis of new collagen or increased collagen breakdown and both. In our study, we found that collagen type I expression was reduced in high glucose-treated HDFs. This implied that the HDFs play a vital role in collagen secretion in diabetic skin. In addition, our subsequent study found that the balance of MMP-2/TIMP-2 and MMP-9/TIMP-1, which can regulate collagen synthesis and decomposition, was disrupted after HDFs were cultured with high glucose. To verify the regulatory relationship among the MMP-2/TIMP-2 and MMP-9/TIMP-1 ratio and skin collagen deposition disorder, rescue experiments were implemented. By inhibiting the activities of MMP2 and MMP9 in diabetic mice, the disorder of collagen deposition was alleviated. Combined with all findings, we have reason to believe that the collagen deposition disorder in diabetic non-injured skin is caused by both decreased synthesis of new collagen and increased collagen breakdown. The imbalance of MMP-2/TIMP-2 and MMP-9/TIMP-1 contributes to the skin disorder of collagen deposition in diabetes patients, providing ideas for managing diabetes skin complications early.

The pathogenesis of diabetic skin lesions is complex and has not yet been fully understood. Some researchers believe that the epidermal structural barrier in the skin of diabetes patients is damaged (22) and prolonged hyperglycemic stimulation and insulin deficiency inhibit keratinocyte proliferation and directional migration, interfering with epidermal differentiation (23–26). In addition, the glycosylation of collagen in the dermis makes the ECM hard and brittle, reducing the mechanical competence of the dermis (27). The glycosylated ECM can regulate the morphology and function of surrounding cells, such as by inducing the differentiation and migration of keratinocytes (28) and reducing the adhesion of dermal fibroblasts and their proliferation and migration (27). In this study, it was found that the skin collagen of diabetes patients become disordered and its density was variable, resulting in aged appearance skin (12), which likely changed the mechanical competence of the dermis. Both the destruction of the epidermal structural barrier and the reduction of the mechanical competence of the dermis impair the mechanical stability of skin; decrease skin resistance to physical, mechanical, chemical, and pathogenic microbial invasion; and increase the risk of skin injury. Although the methods for early diagnosis of skin changes in diabetes patients are limited, it is of great clinical significance to improve awareness for the diagnosis of early non-injured skin (29).

The synthesis and degradation of ECM are primarily regulated by the balance between MMPs/TIMPs that destroys the stability of the ECM and promotes the occurrence of skin injury. The ratio of MMPs/TIMPs is unbalanced in the skin tissue of diabetes patients. The imbalance of MMPs/TIMPs in diabetes may be related to continuous pro-inflammatory and -fibrotic factors being secreted by tissues and cells under high glucose. Conversely, the disrupted balance of MMPs/TIMPs may be caused by the formation of early glycosylation products (30). Generally, MMPs can be classified into four types according to substrate specificity: stromeolysins (MMP-3, -10, and -11); membrane-type MMPs (MT-MMPs, MMP-14–17, -24, and -25); collagenases (MMP-1, -8, -13, and -18), which degrade fibrous collagen into shorter fragments; and gelatinases (MMP-2 and -9), which degrade type IV collagen and gelatin (31, 32). The dermal ECM primarily consists of type I followed by type III collagen. Its stability is greatly affected by the synthesis and decomposition of type I collagen. In addition to being catabolized by collagenase, its decomposed fragments can be further degraded by MMP-2 and -9 (33, 34). Besides, canstatin, a type IV collagen fragment catabolized by MMP-2 and -9, is reported to regulate the migration and secretion of fibroblasts (35). Thus, the function of MMP-2 and -9 also plays a role in regulating the synthesis and decomposition of type I collagen, and their aberrant expression may change the stability of skin collagen.

Increased expression of MMPs has been reported in chronic wounds. Besides, some studies have reported that the expression of MMP-1, -2, and -9 increased in non-injured skin of diabetes patients (12, 36, 37). Unlike these studies, considering the interaction of MMPs and TIMPs, we focused more on its balance. Furthermore, to understand the effect of the disturbance of this delicate balance of MMPs/TIMPs, *in vivo* experiments were conducted on the regaining of this balance in the present study.

Previous studies indicate that MMPs can act multifunctionally in the regulation of inflammation, wound repairing, and wound healing (38, 39). Chronic nonhealing wounds in DM go through multiple stages. The skin goes from normal in the non-diabetes stage to non-injured in the early diabetes stage; the progression is from slight injury to wound formation and finally to nonhealing, accompanied by the persistent abnormal expression of MMPs. The loss of the balance of MMPs/TIMPs inhibits the overexpression of MMPs selectively and can promote the healing process in wounds (40, 41). Similarly, determining whether early intervention of the overexpression of MMPs can prevent or reduce the occurrence of skin injury to reduce the formation of wounds requires further exploration. The present study provides insights into this to some extent.

Although we observed that collagen deposition disorders were alleviated after inhibiting the activity of MMP-2 and -9 in the skin of diabetic mice, the details of this mechanism were not elucidated, which serve as a basis for our future studies. In addition, as a result of experimental condition limitations, there was a lack of morphological and mechanical analyses of disordered collagen fibers in the skin of diabetes patients, limiting our understanding of the stability change of the skin structure to some degree.

## Data Availability Statement

According to national legislation/guidelines, specifically the Administrative Regulations of the People’s Republic of China on Human Genetic Resources (http://www.gov.cn/zhengce/content/2019-06/10/content_5398829.htm, http://english.www.gov.cn/policies/latest_releases/2019/06/10/content_281476708945462.htm), no additional raw data are available at this time. Data of this project can be accessed after an approval application to the China National Genebank (CNGB, https://db.cngb.org/cnsa/). Please refer to https://db.cngb.org/, or email: CNGBdb@cngb.org for detailed application guidance. The accession code CNP0000999 should be included in the application.

## Ethics Statement

The studies involving human participants were reviewed and approved by Wuhan Union Hospital Ethics Committee (REC 08/H1202/137). The patients/participants provided their written informed consent to participate in this study.

## Author Contributions

PZ, CY, and SZ performed experiments. Z-XK and D-XC generated the mice. PZ and CY analyzed data and interpreted results of experiments. Y-QL and QL conceived and designed research, prepared figures, and drafted manuscript. All authors contributed to the article and approved the submitted version.

## Funding

We would like to thank the National Natural Science Foundation of China (No. 81900432 to QL) for funding.

## Conflict of Interest

The authors declare that the research was conducted in the absence of any commercial or financial relationships that could be construed as a potential conflict of interest.

## Publisher’s Note

All claims expressed in this article are solely those of the authors and do not necessarily represent those of their affiliated organizations, or those of the publisher, the editors and the reviewers. Any product that may be evaluated in this article, or claim that may be made by its manufacturer, is not guaranteed or endorsed by the publisher.

## Supplementary Material

Supplementary Figure 1 RT‐qPCR validation of the MMP-2/TIMP-2 and MMP-9/TIMP-1 ratio in skin of diabetic mice. **(A–C)** The expression of MMP-2 and TIMP-2 mRNA in mice skin and the MMP-2/TIMP-2 ratio. **(D–F)** MMP-9 and TIMP-1 mRNA expression in mice skin and the MMP-9/TIMP-1 ratio. The independent experiment was repeated three times. The results are provided as the means ± SEM, **p* < 0.05, ***p* < 0.01 compared with the control.
