# Supplementary figures and images for: The Imbalance of MMP-2/TIMP-2 and MMP-9/TIMP-1 Contributes to Collagen Deposition Disorder in Diabetic Non-Injured Skin
Source: Front Endocrinol (Lausanne). 2021 Oct 27;12:734485. doi: 10.3389/fendo.2021.734485 (PMC8579102; doi:10.3389/fendo.2021.734485)

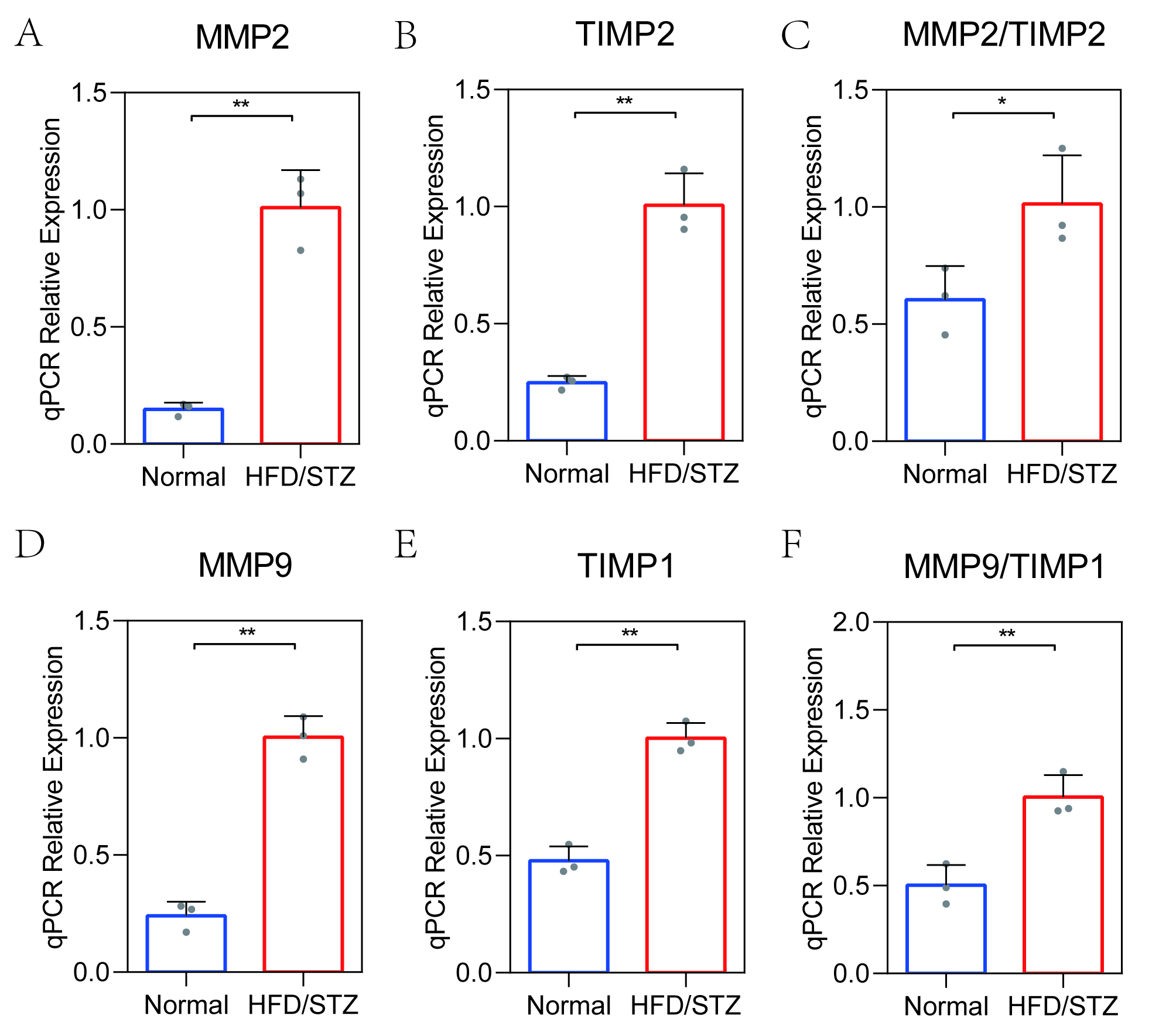

Supplement: Supplementary Figure 1 — RT‐qPCR validation of the MMP-2/TIMP-2 and MMP-9/TIMP-1 ratio in skin of diabetic mice. (A–C) The expression of MMP-2 and TIMP-2 mRNA in mice skin and the MMP-2/TIMP-2 ratio. (D–F) MMP-9 and TIMP-1 mRNA expression in mice skin and the MMP-9/TIMP-1 ratio. The independent experiment was repeated three times. The results are provided as the means ± SEM, *p < 0.05, **p < 0.01 compared with the control. [file Image_1.tif]
